# Point-of-care antimicrobial coating protects orthopaedic implants from bacterial challenge

**DOI:** 10.1038/s41467-021-25383-z

**Published:** 2021-09-16

**Authors:** Weixian Xi, Vishal Hegde, Stephen D. Zoller, Howard Y. Park, Christopher M. Hart, Takeru Kondo, Christopher D. Hamad, Yan Hu, Amanda H. Loftin, Daniel O. Johansen, Zachary Burke, Samuel Clarkson, Chad Ishmael, Kellyn Hori, Zeinab Mamouei, Hiroko Okawa, Ichiro Nishimura, Nicholas M. Bernthal, Tatiana Segura

**Affiliations:** 1 Department of Chemical and Biomolecular Engineering, University of California Los Angeles, Los Angeles, CA United States; 2 Department of Orthopaedic Surgery, Orthopaedic Hospital Research Center, David Geffen School of Medicine at University of California Los Angeles, Los Angeles, CA United States; 3 Weintraub Center for Reconstructive Biotechnology, Division of Advanced Prosthodontics, University of California Los Angeles School of Dentistry, Los Angeles, CA United States; 4 Department of Biomedical Engineering, Neurology, Dermatology, Duke University, Durham, NC United States

**Keywords:** Biomedical materials, Implants, Antimicrobials

## Abstract

Implant related infections are the most common cause of joint arthroplasty failure, requiring revision surgeries and a new implant, resulting in a cost of $8.6 billion annually. To address this problem, we created a class of coating technology that is applied in the operating room, in a procedure that takes less than 10 min, and can incorporate any desired antibiotic. Our coating technology uses an in situ coupling reaction of branched poly(ethylene glycol) and poly(allyl mercaptan) (PEG-PAM) polymers to generate an amphiphilic polymeric coating. We show in vivo efficacy in preventing implant infection in both post-arthroplasty infection and post-spinal surgery infection mouse models. Our technology displays efficacy with or without systemic antibiotics, the standard of care. Our coating technology is applied in a clinically relevant time frame, does not require modification of implant manufacturing process, and does not change the implant shelf life.

## Abstract (web summary)

Implant infection is the most common mode of joint replacement failure with serious complications. Here, the authors report on the in vivo application of a prophylactic coating technology that can incorporate a range of antibiotics and be applied in the operating room prior to implantation.

## Introduction

Orthopaedic surgery is premised on the concept of restoring mechanical stability for the patient, thereby enhancing function. In nearly every surgery, metallic implants are critical to helping replace arthritic joints, fix unstable fractures, or stabilize a deformed spine. Despite decades of research and widespread advances in perioperative antibiotics, aseptic surgical techniques, and patient optimization modalities, a certain percentage of orthopaedic surgeries continue to develop infections that involve the implant. In total joint replacement surgery, for example, periprosthetic joint infection (PJI) occurs in 1% of primary and 3–7% of revision surgeries^1–6^. These patients unfortunately require repeated revision surgeries, prolonged intravenous antibiotic therapy, and carry a higher risk of 5 year mortality than patients diagnosed with HIV/AIDS, breast cancer, or multiple myeloma^7^. In addition to the devastating outcomes suffered by the patient, implant infections are a massive economic burden on the health system, estimated to cost more than $8.6 billion annually in the United States alone^8,9^.

The difficulty in treating an orthopaedic implant infection stems from the formation of a biofilm by the colonizing bacteria. These extracellular polymeric substances form on the avascular metallic implant and render bacteria impenetrable to the host immune response and systemic antibiotics, reducing their efficacy by 1000-fold^10^. This makes treatment with intravenous antibiotic therapy, normally the primary agent used to combat infection, of limited utility. Absolute efficacy is restricted by the systemic toxicity of doses required to achieve bactericidal activity at the site of infection. Successful treatment of these infections therefore requires removal of the implant, a decision that is not inconsequential for the patient, provider, or payer. In addition, in spine surgery, explantation is often the last resort, as it can destabilize the spine, especially if removed prior to definitive fusion. In order to address this difficult situation, surgeons have recently attempted to increase local antibiotic concentrations by either application of antibiotic powder to the wound prior to closure, or by the addition of antibiotics to the polymethylmethacralate (PMMA) cement that is often used as a mechanical grout to secure implants to bone^11^.

Unfortunately, these improvised local antibiotic applications remain limited in their efficacy. Antibiotic powder application has a short lifetime in the soft tissue (approximately 24–72 h), after which it no longer has any antibacterial effect^12^. While antibiotic-loaded bone cement has shown increased efficacy compared to IV antibiotics, it has a variety of intrinsic weaknesses that limit its use^13^. Since PMMA undergoes an exothermic reaction as it sets, only a narrow list of thermally stable antibiotics can be used. In addition, as the amount of antibiotics added to the PMMA increases, the structural properties of the cement begin to degrade, limiting the antibiotic dose^14,15^. Coupled with the poor release kinetics of antibiotics from PMMA, it is difficult for surgeons to have any confidence that appropriate minimum inhibitory concentrations of antibiotics are being reached in the wound bed for any clinically significant amount of time^11^. Finally, after releasing the antibiotic, the PMMA remains an inert porous material that can paradoxically become a nidus for any persistent infection at the surgical site.

With these deficiencies in mind, newer antimicrobial implant coatings have been developed to help prevent bacterial colonization and subsequent biofilm formation long term. Techniques with a clinical track record include iodine implant coatings in Japan and nanosilver coatings in Europe^16,17^. Although these coatings have shown some suppression of microbial activity in PJI, concerns over toxicity have halted FDA approval in the US^18^. Although other, more traditional coatings incorporating antibiotics are currently under investigation^19–24^, many would require a fundamental change in the implant manufacturing process. This would subsequently necessitate a change in FDA classification from device to combination device, requiring new FDA approval, and imposing a shelf-life for the implant that is likely to be much shorter. For these reasons, most implant coating approaches being explored to date are not commercially viable.

To address this challenge, we endeavored to design a biodegradable implant coating technology that can be applied at the point-of-care in the operating room and does not require modification of the implant manufacturing process. Our coating technology is built on earlier work demonstrating that block copolymers of poly(ethylene glycol) (PEG) and sulfur-containing polymers such as poly(propylene sulfide) (PPS) effectively bind metal surfaces^25^, while also solubilizing hydrophobic drugs^26^. We extended this work by synthesizing branched block copolymers of PEG and PPS and demonstrating that these polymers can self-assemble to form biocompatible viscoelastic gels^27^. Further, we demonstrated that these branched polymers retained their ability to bind to metal surfaces, solubilize antibiotic drugs, and coat orthopaedic implants to prevent implant infection^28^. This coating effectively released antibiotics in a dual mechanism that involved both a sustained passive release and an “active” release in the presence of bacteria. However, the PEG-PPS coating technology was limited in that it necessitated silanization of the implant surface to result in robust and stable binding, needed several coating layers to be applied, and took over 10 h to complete. All of these requirements made this coating technology impractical to execute in the operating room setting^28^.

In response to the limitations of PEG-PPS, we re-designed the sulfur-containing portion of the block copolymer by decreasing the synthetic steps, then removing the need for pre-silanization, multiple layers, and long coating times, while maintaining its ability to self-assemble, solubilize antibiotics, elute antibiotics using a dual passive and active release mechanism and biodegrade in a reasonable time frame. We utilized thiol-ene “click” chemistry to first synthesize a linear analog of PPS, polyallyl mercaptan (PAM), and second, to synthesize an amphiphilic branched block copolymer of PEG and PAM (PEG-PAM) on the implant surface. The second click reaction is performed in the presence of antibiotics, incorporating them into the resulting coating.

We proceed to test this PEG-PAM coating both in vitro and in vivo. In vitro, we examine the ability of the coating to incorporate a variety of different antibiotics, and the efficacy of the coating in the face of a bacterial challenge using two different bacterial strains and two different bacterial killing assays. We subsequently examine the ability of the antimicrobial coating to prevent infection in vivo, using two different mouse models of orthopaedic implant infection, a knee PJI model and a spine implant infection model.

## Results

### One pot neat synthesis of PAM polymer

**Fig. 1:**
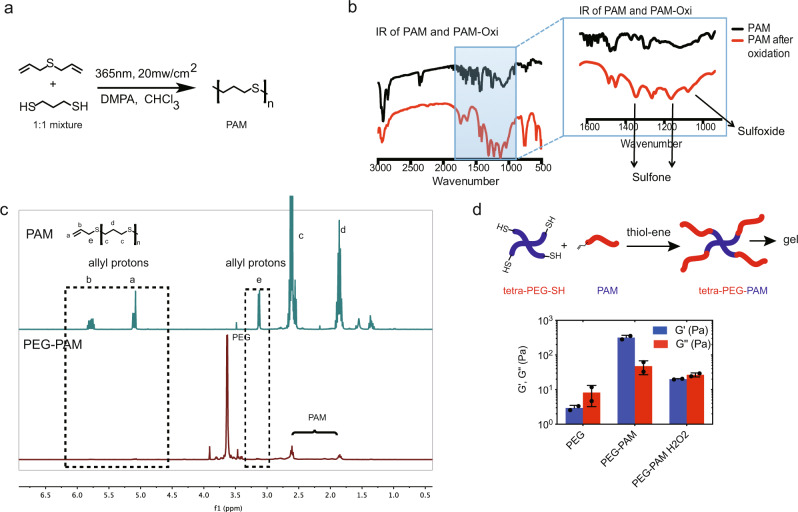
One pot synthesis of polyallyl-mercaptan (PAM) via UV activated thiolene chemistry. **a** Allyl sulfide, 1,3-Dimercaptopropane, and photoinitiator DMPA are dissolved and mixed in chloroform. Upon activation with UV light polymerization generates PAM. **b** FT-IR analysis of PAM polymer and PAM polymer after oxidation with 10% H_2_O_2_, which generates sulfone groups. **c** ^1^H-NMR of PAM and PAM after conjugation of tetra-PEG-SH via UV activated thiol-ene chemistry. Allyl protons are consumed in the reaction with PEG-SH. **d** PEG-PAM at 4 weight % self assembles to form a gel, G’ >G”. Rheometer measurement of modulus of 4% of tetra-PEG-SH, PEG-PAM, PEG-PAM after oxidation in PBS. Data is plotted showing the mean and Standard Deviation. Biological replicates derived from independent experiments are shown as a dot (*n* = 2).

We aimed to design a branched block copolymer that could effectively chemisorb to metal surfaces and self-assemble to form a uniform coating on the metal surface. Although linear PEG-PPS polymers were shown to effectively bind to gold surfaces, we found that branched PEG-PPS polymers were less effective at coating titanium and steel surfaces and required pre-silanization^28^. We reasoned that we could improve chemisorption by using an analog structure to PPS that does not contain a methyl group in every PPS unit, which we thought limited its ability to effectively pack (Supplementary Fig. 1a). To test our reasoning, we synthesized a PAM analog of PPS, which has the same number of carbon units but no methyl side chain (Supplementary Fig. 1b). The synthesis of PAM is based on a “one-pot” thiol-ene copolymerization from a stoichiometric mixture of 1,3 propanedithiol and diallyl sulfide (Fig. 1a). The polymerization was initiated using 365 nm UV light (20 mw/cm^2^) using a 2,2-Dimethoxy-2-phenylacetophenone (DMPA) photoinitiator producing a linear polymer with thiol and/or allyl functional groups at each end depending on if a slight excess of the dithiol or diallyl is used, enabling further functionalization. The reaction can proceed in the presence of atmospheric oxygen and wet solvent (no special drying procedure was used). Thus, the synthesis of PAM is significantly faster and more straightforward than the anhydrous and inert conditions required for anionic polymerization used for PPS synthesis. The results of the synthesis of poly allyl mercaptan (PAM) from the thiol–ene radical copolymerization of the 1,3 propanedithiol and allyl sulfide are presented in Fig. 1a. Size-exclusive chromatography (SEC) analysis reveals that the PAM product has an Mn of 767 MW (Supplementary Fig. 2), which is equivalent to a degree of polymerization (DP) of 10. Additionally, this DP number is also in agreement with the calculation from ^1^H-NMR residue terminal analysis (Supplementary Fig. 1b).

We next examined the ability of PAM to switch from a hydrophobic to a hydrophilic polymer upon oxidation. We used FT-IR to monitor the chemical transformation of thio-ether to sulfone/sulfoxide upon exposure of PAM to oxidation. After oxidation exposure, the PAM FT-IR spectra showed new peaks at 1100, 1300, and 1030 cm^−1^ representative of sulfone and sulfoxide groups, which provides direct proof of thio-ether oxidation (Fig. 1b). Thus, the PAM polymer is oxidation responsive similar to PPS^29^, and can switch from hydrophobic to hydrophilic behavior in the presence of reactive oxygen species^30^.

Although the ability of PEG-PAM to bind gold has no direct relationship to its ability to bind titanium surfaces, PEG-PPS was able to bind to gold and thus we wanted to assess the ability of PAM to bind to gold. Surface plasmon resonance (SPR) was used to confirm the ability of PEG-PAM to chemisorb to gold surfaces. The SPR response was immediate after the deposition of the PEG-PAM copolymer (0.1 mg/ml in methanol) and reached a plateau quickly. Rinsing yielded a second plateau, which was associated with the chemisorbing monolayer on the metal surface (Supplementary Fig. 3). The PEG-PAM dissociation constant (K_D_) was calculated to be ~4.35 μM in water.

### Tetra-PEG-PAM self assembles and oxidizes

After successful preparation of the hydrophobic PAM block, we aimed to synthesize the branch block copolymer of PEG and PAM by grafting the PAM block to a tetra-PEG-SH core using thiol-ene coupling. One mole of tetra-PEG-SH was used for every four moles of PAM. Our ^1^H-NMR results (Fig. 1c) indicated a total disappearance of allyl protons from PAM terminals, which means almost quantitative coupling between tetra-PEG-SH and PAM moieties. Following purification, tetra-PEG-PAM is soluble in water and it forms a hydrogel at 6% w/v with a storage modulus of ~400 Pa and a loss modulus of ~30 Pa (Fig. 1d). To demonstrate that the hydrogel was formed through self-assembly and not through covalent crosslinking during PEG-PAM synthesis, PEG-PAM hydrogels were oxidized using H_2_O_2_. If crosslinking is through covalent bonds, the modulus of the hydrogel should not change significantly; however, if the crosslinking is through self-assembly, the gel would be completely disrupted when the PAM block changes from hydrophobic to hydrophilic, and the storage modulus would be lower in value than the elastic modulus. Upon exposure to H_2_O_2_, the mechanical properties of the PEG-PAM hydrogel were similar to that of PEG polymer, indicating that PEG-PAM forms a hydrogel through self-assembly.

### In situ tetra-PEG-PAM synthesis effectively coats metal surfaces

**Fig. 2:**
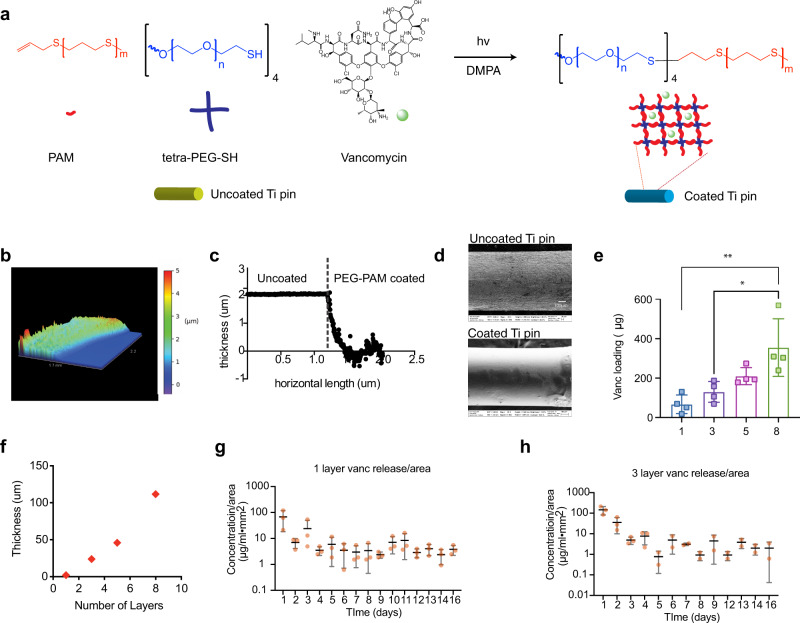
PEG-PAM coating can be UV cured directly on to metal surfaces. **a** PAM, PEG-SH, and photoinitiator DMPA are dissolved in chloroform. The antibiotic (vancomycin for example) is also dissolved in chloroform. The surfaces (pin or flat slide) are submerged in the coating solution and excess reagents removed by gravity, the surface is then exposed to UV light to induce PEG-PAM coupling. **b** 3D profilometer scan of PEG-PAM coated titanium surface. **c** A horizontal and vertical measurement of coating thickness using the 3D profilometer scan reveals a coating thickness of 2 µm. **d** SEM images of uncoated and PEG-PAM/Vanc coated titanium pins show a change in surface roughness as a result of the coating process. **e** Multiple layer deposition of PEG-PAM polymer coatings (3 Layers, 5 Layers, and 8 Layers) with increased thickness and increased loading amount of vancomycin (*n* = 4). **f** The thickness of 1, 3, 5, 8 layers of PEG-PAM coatings on the titanium surface (*n* = 1). **g** In vitro release of vancomycin from single layer PEG-PAM coating (*n* = 3). **h** In vitro release of vancomycin from 3 layers PEG-PAM coating (*n* = 3). Statistics in (**e**) are an ordinary one-way ANOVA with a Tukey Multiple comparison test. **p = 0.002 and *p = 0.012. Data is plotted showing the Mean and Standard Deviation. Biological replicates derived from independent experiments are shown in each panel as a dot.

Our goal was to use tetra-PEG-PAM mixed with antibiotics to effectively coat orthopaedic implant surfaces and prevent infection. However, the fact that PEG-PAM has high viscosity in both aqueous and organic solvents and forms a hydrogel at concentrations over 5% w/v complicates the coating. Since high viscosity and gelation occur only when tetra-PEG and PAM are conjugated to form an amphiphilic branched co-polymer, we reasoned that we could avoid working with this highly viscous solution and effectively coat the implant by applying a solution of tetra-PEG and PAM precursors prior to in situ conjugation to form tetra-PEG-PAM (Fig. 2a). Titanium wires were submerged in a solution containing a mixture of PAM and tetra-PEG-thiol dissolved in chloroform, resulting in a final concentration of 6% w/v of tetra-PEG-PAM polymer. The titanium wires were subsequently irradiated under UV light (365 nm, 20 mw/cm^2^) for 5 min to induce the formation of tetra-PEG-PAM directly on the implant surface and the solvent was subsequently evaporated under vacuum. The drying process can also be accomplished through passive evaporation at the bench surface or through flowing nitrogen gas over the implant surface. All of these approaches are expected to be OR and surgery compatible. The resulting coated implant was analyzed using scanning electron microscopy (SEM), elemental analysis, and gross images using a rhodamine-labeled coating to assess the chemical composition and coating microstructure of the surface. SEM and gross images show the coating covering the implant surface changing it from a rough appearance to a smooth appearance (Supplementary Fig. 4a, b). Elemental analysis revealed an increase in sulfur concentration from 0-wt% to 2-wt% and a reduction in chlorine content to 99.6% relative to the coating solution, consistent with the incorporation of PAM and removal of solvent on the implant surface (Supplementary Fig. 4c, 5). To estimate the coating thickness, mock titanium-coated ultra-flat gold slides were used and analyzed with AFM and 3D profilometer (Fig. 2b, c, Supplementary Fig. 4d, e). AFM and 3D profilometer results showed clear differences between coated and uncoated areas in both roughness and thickness. The thickness of the tetra-PEG-PAM layer was found to be 2 µm.

### In situ PEG-PAM antibiotic coating as an antimicrobial strategy

**Fig. 3:**
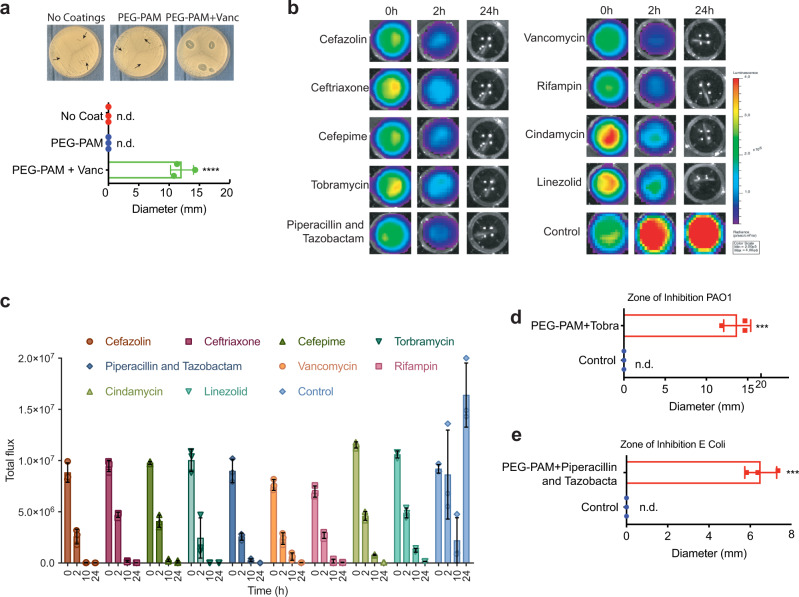
In vitro evaluation of antibiotic loaded PEG-PAM coatings. **a** Representative ZOI images of titanium pins with no coatings, PEG-PAM coating only, PEG-PAM coating with vancomycin against *S. aureus*. ZOI diameter measurement of titanium pins with no coatings, PEG-PAM coating only, PEG-PAM coating with vancomycin. **b** Representative in vitro *S. aureus* bioluminescence on 96 wells plate with PEG-PAM coatings containing different antibiotics, and the control group is uncoated pins. **c** Quantification of in vitro *S. aureus* bioluminescence on 96 wells plate with PEG-PAM coatings containing different antibiotics and the control group is uncoated pins. **d**, **e** ZOI diameter measurement of titanium pins with PEG-PAM coating only, PEG-PAM coating with Tobramycin against PAO1, with PEG-PAM coating only, PEG-PAM coating with Piperacillin and Tazobactam against *E. coli.* **f** Quantification of in vitro *S. aureus* bioluminescence of established biofilm on 96 wells plate before and after PEG-PAM vancomycin (PPV) coatings. Statistics in (**a**) are an ordinary one-way ANOVA with a Tukey Multiple comparison’s test **p < 0.0001. Statistics in (**d**) and (**e**) are two-tailed Student’s *t*-test ***P < 0.001. Data is plotted showing the Mean and Standard Deviation. Biological replicates derived from independent experiments are shown in each panel as a dot (*n* = 3 for all panels).

Antibiotics are introduced into the coating by mixing the coating precursors, tetra-PEG and PAM, with the desired antibiotic prior to coating the implant (Fig. 2a). Since the “thiol-ene” photoconjugation in solution does not release heat, a wide range of antibiotics can be incorporated into the coating. Utilizing vancomycin (Vanc), one of the most commonly used antibiotics after orthopaedic surgery to prevent infection, we studied the parameters that affect antibiotic loading, such as the role of tetra-PEG-PAM concentration in the coating and sequential coating layers. SEM images of coated pins that contained Vanc displayed similar smooth morphology as those with PEG-PAM only (Fig. 2d). Utilizing PEG-PAM from 2% to 20% w/v in the coating solution resulted in an increased concentration of Vanc loaded with increasing percent of the loaded polymer, ranging from 30–50 µg/mm^2^ (Supplementary Fig. 6). Similarly, undergoing the coating process several times results in an increase in Vanc loading with each new layer, ranging from 100–300 µg/mm^2^ for 1 to 8-layers respectively (Fig. 2e). The thickness of the coating was again estimated using flat titanium-coated glass slides and 3D profilometry and increased with each additional layer added reaching 100 µm coating for 8-layers (Fig. 3f). Vanc release in a PBS bath shows that the concentration of Vanc in solution increased over time and was above the MIC (0.5 μg/mL for Xen 36) for 14 days (Fig. 2g). Pins that were coated with 3 layers of PEG-PAM + Vanc resulted in concentrations above the MIC for 17-days (Fig. 2h).

To measure in vitro antimicrobial activity against *S. aureus*, we performed zone of inhibition (ZOI) assays with our PEG-PAM coated titanium pins with/without Vanc. Vanc loaded pins with one layer of 6% w/v coating (60 µg/pin) were placed on top of *S. aureus* (Xen36) agar plates (Fig. 3a). Distinctive dead zones were observed on the plates containing tetra-PEG-PAM/Vanc coated pins, while uncoated pins and tetra-PEG-PAM pins showed no dead zone. Quantification of the ZOI area showed a statistically increased area for the Vanc containing group (Fig. 3a).

The ability to effectively incorporate a variety of antibiotics was confirmed by coating separate implants each with one of 8-different clinically relevant antibiotics including ceftriaxone, cefazolin, cefepime, tobramycin, piperacillin and tazobactam, clindamycin, linezolid, and rifampin. All of these antibiotic-loaded titanium pins were used to verify the ability to inhibit *S. Aureus* growth in vitro (Fig. 3b, c). The coated pins were incubated in a solution of bioluminescent *Staphylococcus aureus* Xen36, which contain a bioluminescent lux operon construct integrated into a stable bacterial plasmid that naturally produces a blue-green light emitted only by metabolically active bacteria. Bioluminescence readings were taken at 0, 2, 8, and 24 h after bacteria inoculation. The cefazolin, tobramycin, piperacillin and tazobactam, and rifampin groups had almost no bioluminescent signal after 8 h, which indicated an efficient inhibition of *S. aureus* growth from antibiotics released from the coated pins (Fig. 3b, c). Quantification revealed a steady decrease in bioluminescence reaching baseline levels by 24 h. In contrast, pins containing only tetra-PEG-PAM resulted in a significant bioluminescence increase by 24 h (Fig. 3b, c). Antibiotic-coated implants were also challenged with other infectious bacterial strains, including *Pseudomonas aeruginosa* PAO-1 and *Escherichia Coli* RR1 with ZOI assays. With the appropriate selection of antibiotics in the PEG-PAM coating (tobramycin for PAO-1 and piperacillin and tazobactam for RR1), distinctive dead zones were observed in the plate containing tetra-PEG-PAM/antibiotic coated pins, while tetra-PEG-PAM pins without antibiotic showed no dead zone (Fig. 3d, e).

### PEG-PAM coating does not inhibit osseointegration long-term

**Fig. 4:**
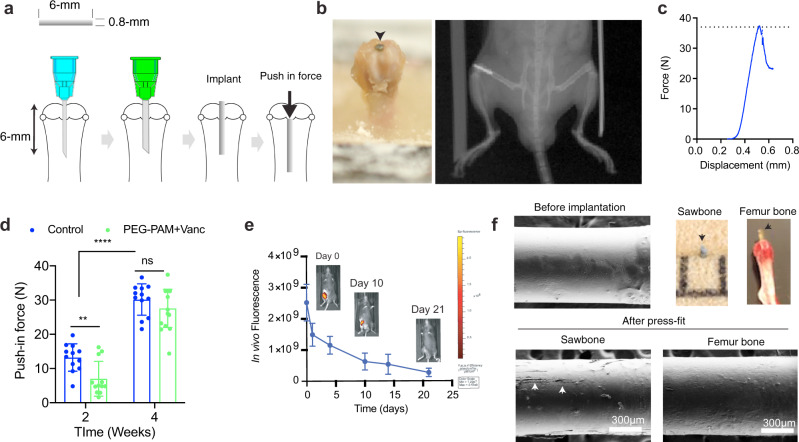
PEG-PAM coating does not prevent long term osseointegration. **a** Schematic of the implant push-in test. Ti implants (0.8 mm diameter and 6 mm length) were prepared from K-wires and both edges were flattened. **b** The distal femur of two groups of mice (24 in each group) were implanted with either a Ti implant that was coated with PEG-PAM/Vanc or a control untreated Ti implant. **c** At 2- and 4-weeks post-implantation, 12 mice from the PEG-PAM/Vanc group and 12 mice from the control group were euthanized and femurs were harvested. A push-in test was performed and the load (N) to displacement was measured as the implant push-in value. The maximal push-in force (shown with a dotted line in **c**) is then plotted for all the biological replicates. **d** In both the control and the PEG-PAM/Vanc coated implant group there was a statistical increase in the mean load to displacement from 2–4 weeks. While the mean load to displacement was significantly lower in the PEG-PAM/Vanc coated group at 2 weeks, there was no difference between the groups 4-weeks post-implantation. Each animal is represented by a dot and were considered biological replicates. Biological replicates are shown (*n* = 12) and data are plotted as the mean ± standard deviation. **e** In vivo biodegradation of PEG-PAM coating labeled with Cy5.5 maleimide using in vivo fluorescence imaging (*n* = 3). The fluorescence scale shown was obtained directly from the IVIS imaging scanner. Data is plotted showing the Mean and Standard Deviation. **f** The PEG-PAM/Vanc coated Ti implants were press-fit into PCF 10 Sawbones block and into cadaver mouse femurs (color images in this panel). The implants were subsequently retrieved and examined by SEM to characterize the mechanical stability of the implant. Characteristic surface abrasions on the PEG-PAM/Vanc coating can be seen and denoted by a white arrow. Statistics in (**d**) are a two-way-repeated measure-ANOVA with multiple comparisons post-test. The significance between treatment groups **p = 0.0061.

Implant surfaces are carefully designed for optimal biocompatibility and osseointegration. Thus, any coating technology must demonstrate that osseointegration is not compromised. To evaluate the effect of the PEG-PAM/Vanc coating on osseointegration, push-in-force measurements were used. The distal femur of 24 mice were implanted with an untreated control Ti implant and 24 mice were implanted with a Ti implant coated with PEG-PAM/Vanc. The technique for implantation was the same as that used in the mouse model of knee PJI, except no bacteria was used for this experiment (Fig. 4a–c, Supplementary Fig. 9)^9^. This animal model was developed by practicing orthopaedic surgeons specifically as a small animal screen for the evaluation of translational technologies. Briefly, a sterile titanium pin (6 mm length × 0.8 mm diameter) is placed retrograde, from the knee joint into the femoral canal by first generating a channel using a 25-gauge and then a 21-gauge needle, consistent with the clinical practice of reaming. At 2- and 4-weeks following implantation, 12 mice from each group were euthanized and the femurs were harvested. The mechanical withholding strength was measured by pushing the implant in a retrograde fashion into the femoral canal using a custom-made stainless-steel pushing rod mounted on a 1000 N load cell (Instron, Canton, MA). The axial load on the implant was applied at a crosshead speed of 1 mm/min, and the maximum load (N) to displacement was measured as the implant push-in value (Fig. 4c)^31^.

We found that in both the control and the PEG-PAM/Vanc coated implant group there was a statistical increase in the mean load to displacement from 2–4 weeks (Fig. 4d). In the control group, the mean load to displacement increased from 13.2 N (standard deviation (SD) 4) to 30.2 N (SD 4.6) while in the PEG-PAM/Vanc coated group the mean load to displacement increased from 7 N (SD 5.1) to 27.6 N (SD 5.6). The mean load to displacement was statistically significantly lower in the PEG-PAM/Vanc coated group at 2 weeks, but there was no difference between the groups 4-weeks post-implantation. This data suggests that the process of osseointegration was ongoing in both groups between 2 and 4 weeks and that the PEG-PAM/Vanc coating does not inhibit the establishment of osseointegration by 4 weeks post-implantation.

To assess whether the delay in osseointegration corresponds to the presence of the coating, we assessed the in vivo degradation rate of PEG-PAM coating using the same PJI mouse model. In vivo biodegradability of PEG-PAM coating was assessed using non-invasive imaging through labeling PEG-PAM with Cy5.5. Following implantation, the decay of fluorescent signal was monitored in the mice for 3 weeks. We found a steady fluorescence decrease in signal over time with no detectable level at 3-weeks (Fig. 4e). Given that Cy5.5 last more than 4 weeks in vivo^32^ we conclude that coating degradation, rather than fluorophore degradation, is responsible for the observed decrease and that our coating is largely resorbed by 3-weeks. These results are consistent with the observed osseointegration and with the notion that the implant surface retains its ability to undergo osseointegration after coating resorption.

### PEG-PAM coating can withstand mechanical forces during implantation

Though not all orthopaedic implant surgeries impose mechanical shear to the implant surface, the press-fit technique in the PJI model does and thus, we set to determine to what extent the coating sheared after press-fit implantation. Two distinct methods were used to qualitatively evaluate the mechanical stability of the PEG-PAM/Vanc coating following press-fit implantation. In each case, 6 Ti implants were coated with PEG-PAM/Vanc. The first method employed a synthetic bone mimic (10 pounds per cubic foot (PCF) polyurethane foam block, Sawbones). 10 PCF was selected as it has similar tensile modulus and compressive strength as the trabecular bone in the human distal femur^33,34^. We compressed the flat surfaces of two Sawbones blocks together using lag screws. The potential space between the two blocks was then reamed first with a 25- and then a 21-gauge needle as per our implantation protocol and the coated implants were inserted by hand (Fig. 4f, Supplementary Fig. 7). Following insertion, the screws in the blocks were removed, the blocks separated, and the implants carefully lifted from the blocks and imaged via scanning electron microscopy (SEM). Using this method, the force on the coating was only felt during insertion, thus creating a more realistic model of clinical reality in which the bone is first reamed and then the implant is inserted and left in place.

The second set of 6 coated implants were press-fit in a retrograde fashion into freshly harvested cadaver mouse femurs, and then pulled out from the direction in which they had been inserted (Supplementary Fig. 8). Following insertion and removal, the implants were imaged via SEM. The insertion site in the intercondylar notch was sequentially reamed first with a 25- and then a 21-gauge needle prior to implant insertion (Supplementary Fig. 9). Unlike the Sawbones technique described above, in the cadaver femur technique the coating experienced both the force of insertion and removal. The post-implantation and removal SEM images for both the Sawbones and cadaver mouse femur methods showed that the coating was largely intact, with only rare surface abrasions (Fig. 4f, Supplementary Fig. 7, 8).

### Antibiotic loaded Tetra-PEG-PAM coating effectively protects the implant surface from bacterial challenge in a PJI model

**Fig. 5:**
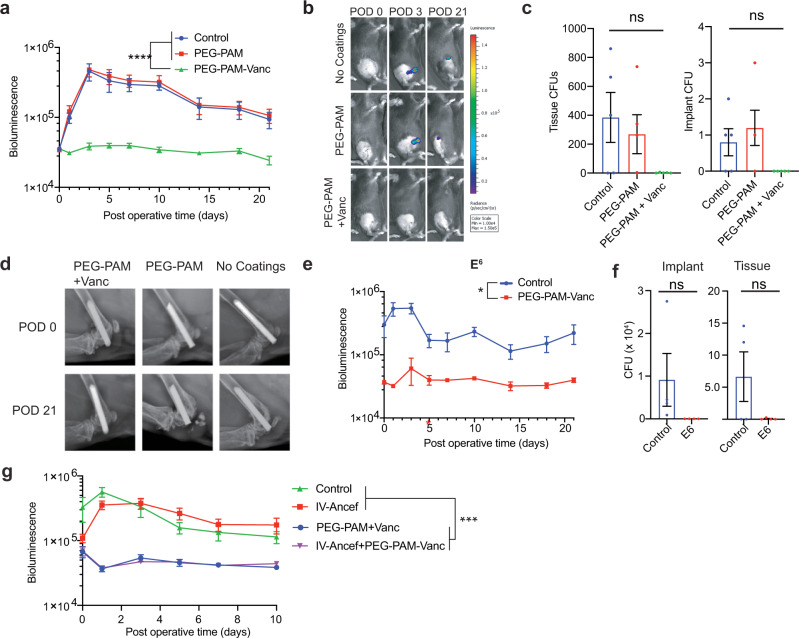
PEG-PAM coatings loaded with vancomycin prevent implant infection. **a** Postoperative in vivo *S. aureus* bioluminescence signal (mean maximum flux [photons/sec/cm^2^/sr] (logarithmic scale, *n* = 15 for no coatings group, *n* = 14 for PEG-PAM only group, *n* = 14 for PEG-PAM-Vanc group). **b** Representative in vivo *S. aureus* bioluminescence on a color scale overlaid on grayscale images of mice (**c**) Quantification of colony-forming units (CFUs) cultured from surrounding tissue and implant (*n* = 5 for no coating group, PEG-PAM group, and PEG-PAM + Vanc group). **d** Representative radiographs from the no coatings, PEG-PAM, PEG-PAM + Vanc groups of POD 0 and 21. **e** Postoperative in vivo S. *aureus* bioluminescence signals with 1.0 × 10^6^ CFUs loadings of bacterial challenge with PEG-PAM/Vanc coating, *n* = 4 for the control group, *n* = 6 for PEG-PAM/Vanc group for the bioluminescence measurement, *n* = 4 for the control group, *n* = 4 for PEG-PAM/Vanc group in 1.0 × 10^6^ loading group in CFU counting experiment. **f** Postoperative in vivo *S. aureus* bioluminescence signal comparison of PEG-PAM/Vanc coating, IV injection/ancef, IV injection/ancef with PEG-PAM/Vanc and control groups. (*n* = 8 for control group, *n* = 6 for PEG-PAM/Vanc group, *n* = 6 for IV-ancef group, *n* = 6 for IV-ancef + PEG-PAM/Vanc group). Statistics in (**a**, **e**, and **f**) are a two-way-repeated measure-ANOVA with multiple comparisons post-test. The significance between treatment groups was ****p < 0.0001 For (**a**), was ***p = 0.003 for (**e**), and ***p = 0.0004 for (**g**). Statistics in (**c** and **f**) are not significant using an ordinary one-way ANOVA and *t*-test respectively. Data is plotted showing the Mean and Standard Deviation for bar plots and Standard Error of the Mean for time longitudinal plots.

We next evaluated if our implant coating technology could effectively prevent infection after periprosthetic joint infection using the knee PJI model. Bioluminescent *Staphylococcus aureus* Xen36 (10^3^ bacterial light units) was used to inoculate the intra-articular portion of the metal pin in the joint space to induce the formation of a controlled infection that can be non-invasively quantified using the Xenogen in vivo imaging system (IVIS Lumina II, PerkinElmer, Hopkinton, MA). We tested joints treated with implants that had no coating, PEG-PAM or PEG-PAM/Vanc. In the mice that received implants with one layer of 6% w/v tetra-PEG-PAM/Vanc coating the bioluminescent signals were significantly lower (*p* < 0.05), compared to mice with implants that were not coated or were treated with the tetra-PEG-PAM coating alone (Fig. 5a, b), indicating that the tetra-PEG-PAM polymer alone has no antibacterial properties and cannot protect the implant surface. The Vanc coated implants had a bioluminescence signal that was not above baseline from post-operative day (POD) 1 onward, suggesting eradication of infection below the level of detection by noninvasive imaging. However, bacteria could still be present at low levels at the implant surface and surrounding tissue.

To assess the presence of bacteria at the implant surface and tissue, at POD 21, the implants and surrounding tissue were retrieved, and the number of colony-forming-units (CFUs) was assessed (Fig. 5c). As expected, animals that received infected implants had 100% of the implants and tissue grow out of bacteria. Similarly, animals that received PEG-PAM-only implants had 100% of the implants and tissue grow out of bacteria, again showing that PEG-PAM coating itself is not antimicrobial nor protects the surface from infection. Animals that received implants that were coated with PEG-PAM/Vanc resulted in none (0%) of the implants containing CFUs, showing that PEG-PAM/Vanc coating effectively and completely protects the implant surface from infection.

Periprosthetic osteolysis is a major complication of orthopedic joint infections, where bone loss occurs and the implant loosens due to the local inflammatory response to infection. Animals treated with implants coated with tetra-PEG-PAM alone showed a dramatic degree of periprosthetic osteolysis that became evident by POD 21 (Fig. 5d). In contrast, animals treated with PEG-PAM/Vanc coated implants showed no detectable radiographic periprosthetic osteolysis (Fig. 5d), consistent with the high efficacy of these coatings in preventing bacterial replication and subsequent host immune response around the implants and the surrounding bone and soft tissue. To examine the effect of the PEG-PAM/Vanc antibiotic implant coating on the microscopic anatomy of the distal femur, histologic sections of the tissue were evaluated (Supplementary Fig. 10a). Uncoated pins, as well as pins coated with PEG-PAM without antibiotics, demonstrated morphologic changes in the bone and surrounding joint tissue. These changes included increases in the size of the distal femur and changes to the normal bony architecture, as well as synovial hyperplasia and an inflammatory cell infiltrate. These alterations were less pronounced in distal femoral tissue from PEG-PAM/Vanc coated pins. Taken together, these findings show that the tetra-PEG-PAM/Vanc coating protected the implant surface and surrounding tissue from infection and the resulting inflammatory reaction that can lead to periprosthetic osteolysis.

To challenge the coating further, the implants were infected with orders of magnitude higher numbers of bacteria. We infected the implants with 10^4^, 10^5^, 10^6^ bacterial light units instead of 10^3^ as previously done. Surprisingly, in 10^4^ and 10^6^ bacterial challenge conditions, one layer of tetra-PEG-PAM/Vanc effectively protected the implant surface from infection with no implants containing CFUs at day 21-post implantation (Fig. 5e, Supplementary Fig. 10b). However, animals receiving 10^5^ bacteria a single animal contained CFUs at both the implant and surrounding tissue (Supplementary Fig. 10c), indicating some variability in the coating or surgical procedure. Nevertheless, completely preventing infection at 10^3^, 10^4^, and 10^6^ bacterial challenge conditions demonstrates that our PEG-PAM/Vanc implant coating is able to completely protect the implant surface from infection.

Given that it is likely that this technology would be applied in conjunction with the current standard of care, IV antibiotics, we compared IV antibiotic delivery alone or in combination with PEG-PAM/Vanc coating. Utilizing a mouse-equivalent dose of the standard of care IV delivered antibiotic resulted in a higher bioluminescence signal than the PEG-PAM/Vanc group from post-operative day 0 to day 10. We have performed studies directly comparing IV-delivered antibiotics and our PEG-PAM/Vanc coating. Our results demonstrate that implants coated with PEG-PAM/Vanc were statistically more protected from infection than the standard of care. Bioluminescence curves of animals treated with PEG-PAM/Vanc coated implants and the combined treatment of PEG-PAM/Vanc coated implants plus IV antibiotics were indistinguishable from each other (Fig. 5f), suggesting that the coating alone effectively prevents infection.

### Loading of multiple antibiotics in PEG-PAM coating

**Fig. 6:**
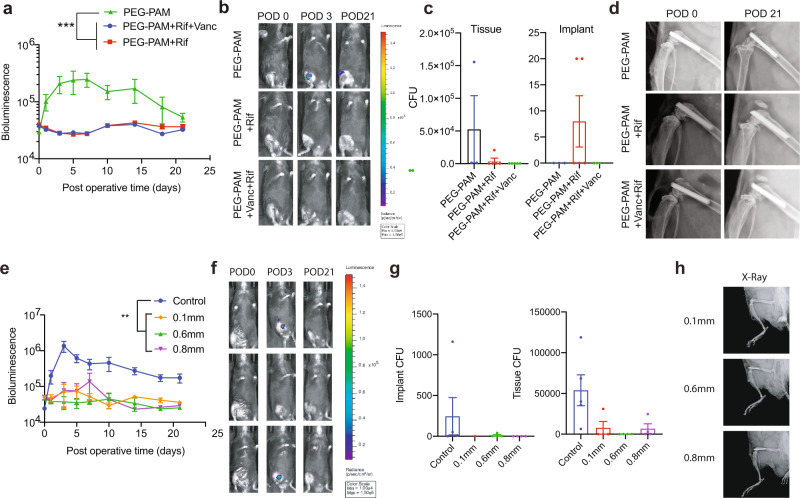
In vivo evaluation of PEG-PAM coatings with combination of Rifampin and vancomycin. (**a**) Postoperative in vivo *S. aureus* bioluminescence signals (mean maximum flux [photons/sec/cm^2^/sr] (logarithmic scale) (*n* = 6 for PEG-PAM only group, *n* = 6 for PEG-PAM + Vanc group, *n* = 6 for PEG-PAM + Vanc + Rif). **b** Representative in vivo *S. aureus* bioluminescence on a color scale overlaid on grayscale images of mice. **c** Representative radiographs from the PEG-PAM, PEG-PAM with rifampin, PEG-PAM with vancomycin, and rifampin groups of POD 0 and 21. **d** Quantification of colony-forming units (CFUs) cultured from surrounding tissue and implant. In vivo evaluation of PEG-PAM coatings on different sized stainless-steel pins with vancomycin were evaluated. **e** Postoperative in vivo *S. aureus* bioluminescence signals (mean maximum flux [photons/sec/cm^2^/sr] (logarithmic scale, *n* = 5 for control group, *n* = 5 for 0.6 mm group, *n* = 5 for 0.8 mm group, *n* = 5 for 0.1 mm group). **f** Representative in vivo *S. aureus* bioluminescence on a color scale overlaid on grayscale images of PEG-PAM with vancomycin and vanc groups of POD 0, 3, and 21. **g** Quantification of colony-forming units (CFUs) cultured from surrounding tissue and implant. (*n* = 5 for the control group, *n* = 4 for the 0.6 mm group, *n* = 4 for the 0.8 mm group, *n* = 4 for the 0.1 mm group). **h** X-ray images of implants (0.1, 0,6, and 0.8 mm). Statistics in (**a**, **e**) are a two-way-repeated measure-ANOVA with multiple comparisons post-test. The significance between treatment groups was ***p = 0.007 for (**a**) and ***p = 0.0001 for (**e**). Statistics in (**c** and **g**) are not significant using an ordinary one-way ANOVA. Data is plotted showing the Mean and Standard Deviation for bar plots and Standard Error of the Mean for time longitudinal plots.

In practice, there is often a need to introduce multiple antibiotic types into a single implant to establish broad-spectrum antimicrobial therapy. Multiple types of bacteria may be present, requiring multiple types of antibiotics for adequate treatment. Multiple antimicrobial agents can also be used to ensure that the bacteria are completely eliminated, reducing the risk of generating antibiotic-resistant bacterial strains. Thus, we investigated the incorporation of two antibiotics into the same implant coating using PEG-PAM. Implants were coated with rifampin or a mixture of rifampin and vancomycin using the same coating procedure as used above and resulted in 37.8 µg/implant for rifampin in rifampin-only coatings and 28.0 µg/implant rifampin and 12.8 µg/implant vancomycin for the implants that contained both antibiotics. Both implants coated with tetra-PEG-PAM/Rifampin and tetra-PEG-PAM/Vancomycin/Rifampin effectively protected the implant surface from infection (Fig. 6a, b), decreased the number of CFUs on and around the implant surface (Fig. 6c), and protected the implant from periprosthetic osteolysis (Fig. 6d).

### Loading of vancomycin in PEG-PAM coating on different sized stainless-steel implants

In orthopaedic surgery, implants are usually diversified with different metals of multiple sizes. Thus, we explored the compatibility of our coating technology using different sized stainless-steel implants (0.1, 0.6, and 0.8 mm). All these implants were coated with vancomycin using the same procedure and were examined in our mouse model of knee PJI. Our in vivo results indicated these implants coated with tetra-PEG-PAM/Vanc effectively protected the implant surface from infection (Fig. 6e, f), decreased the number of CFUs on and around the implant surface (Fig. 6g), and protected the implant from periprosthetic osteolysis (Fig. 6h).

### Antibiotic loaded PEG-PAM coating effectively protects the implant surface from bacterial challenge in spine implant infection model

**Fig. 7:**
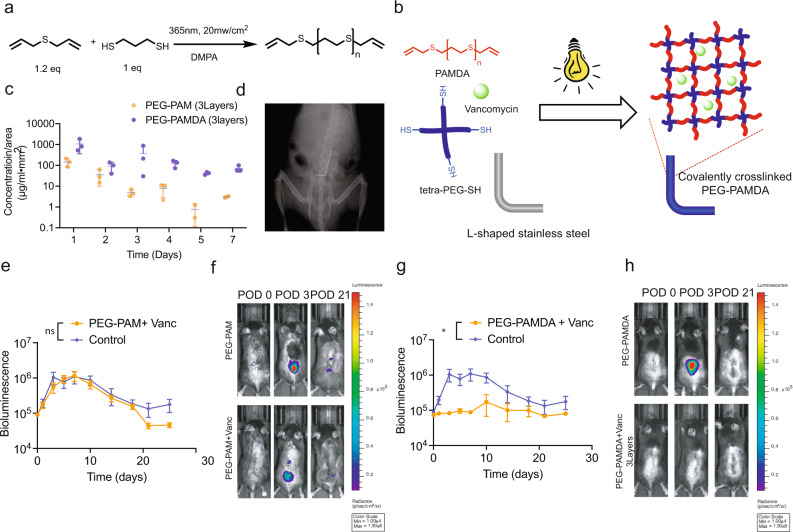
In vivo evaluation of PEG-PAMDA coatings on spine surgery model with vancomycin. **a** Synthetic scheme for PAMDA synthesis. **b** Chemical crosslinking mechanism of tetra-PEG-SH and PAMDA. **c** In vitro release of vancomycin from PEG-PAM single layer coating and 3 PEG-PAMDA layers coating. **d** Representative X-ray image of spine surgery model (**e**) Postoperative in vivo *S. aureus* bioluminescence signals (mean maximum flux [photons/sec/cm2/sr] (logarithmic scale) of single-layer PEG-PAM only and PEG-PAM with vancomycin on stainless spine implant. (*n* = 3 for PEG-PAM group, *n* = 3 for PEG-PAM + Vanc group). **f** Representative in vivo *S. aureus* bioluminescence on a color scale overlaid on grayscale images of PEG-PAM with vancomycin and PEG-PAM only groups of POD 0, 3, and 21. **g** Postoperative in vivo *S. aureus* bioluminescence signals (mean maximum flux [photons/sec/cm2/sr] (logarithmic scale)) of 3 layers PEG-PAMDA only and 3 layers PEG-PAMDA with vancomycin on stainless spine implant (*n* = 6 for 3 layers PEG-PAMDA group, *n* = 6 for 3 layers PEG-PAMDA + Vanc group). **h** Representative in vivo *S. aureus* bioluminescence on a color scale overlaid on grayscale images of 3 layers PEG-PAMDA with vancomycin and 3 layers PEG-PAMDA only groups of POD 0, 3, and 21. Statistics in (**c**, **e**, **g**) are a two-way-repeated measure-ANOVA with multiple comparisons post-test. The significance between treatment groups was not significant for (**c**) and (**e**) and *p = 0.0116 for (**g**) Data is plotted showing the Standard Error of the Mean for time longitudinal plots.

To demonstrate the versatility of our coating technology, we explored the possibility of transferring the coating process to a spine implant infection model^35^. Besides PJI, spine implant infection is another devastating complication that occurs in 2–8% of all elective spine surgeries. Briefly, an L-shaped stainless-steel pin (0.1 mm) is placed through the L4 spinous process and laid cranially along with the posterior spinal elements. Bioluminescent *Staphylococcus aureus* Xen36 is used to inoculate the bend of the metal pin to induce the formation of a controlled infection that can be non-invasively quantified using the Xenogen in vivo imaging system (IVIS Lumina II). We performed the same PEG-PAM coupling with vancomycin on the 0.1 mm “L” shaped stainless-steel implant. Our in vitro release experiment indicated an elution of vancomycin for 1 week above MIC. However, the coating was ineffective in preventing infection resulting in the same level of infection as PEG-PAM coating alone. (Fig. 7e, f).

To overcome this limitation and deliver more antibiotics in the spine surgery model, we designed and synthesized a more stable polymer network: PEG-PAMDA (polyallyl mercaptan diallyl), which is covalently cross-linked by a thiol-ene click reaction between tetra-PEG-SH and PAMDA. In addition, based on our previous results, we increased the coating layer from 1–3 to maintain 3 times more antibiotic (352 ug) loading compared with the single-layer PEG-PAM coating (140 ug). (Fig. 7c) Animals treated with this improved PAMDA/Vanc coated implants had significantly decreased bacterial infection when compared to PAMDA coating alone (Fig. 7g–h). These results indicate that a relatively simple modification of the coating, that allows for further crosslinking, is more effective for preventing infection in spinal implants.

As mentioned, the off-label application of antibiotic powder to the wound is used at times to prevent infection. This is often the case for spine surgeries. We recently assessed and published on the use of vancomycin powder to prevent infection^35^. In these studies, we find that vancomcyin powder completely suppresses bioluminescence signal; however, 20% of the animals have persistent infections despite the large amounts of vancomycin powder used of 2, 4, and 8 mg per wound.

### Demonstration of PEG-PAM coating on a clinically used implant in operating room

**Fig. 8:**
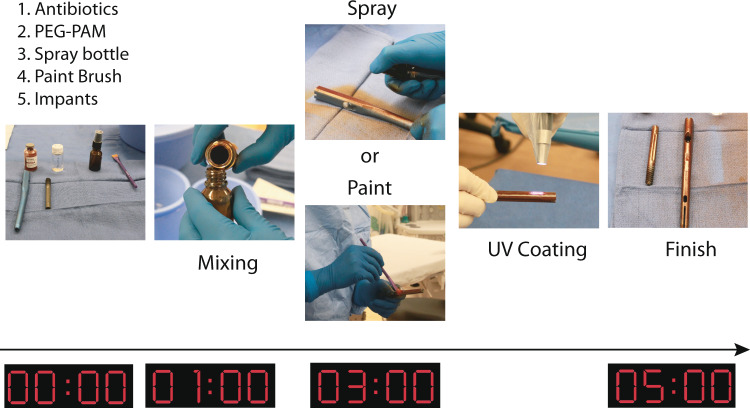
Demonstration of PEG-PAM coating technology of hip implant in operating room. PEG, PAM, photoinitiators are pre-weighted into a container. Chloroform is supplied at the appropriate volume to make a 6% solution of polymers. Pre-weighted antibiotics are thoroughly mixed with the dissolved polymers and either added to a sprayer apparatus or in a container to be painted. The polymer + antibiotic mix is then evenly applied to the implant surface. In this example, the antibiotic is red and easily seen on the implant surface. If needed a biocompatible dye can be added to the mixture for visualization. Following complete coverage, the implant is UV cured for two minutes.

To further demonstrate the potential applicability of our coating technology, we performed the PEG-PAM coating process in the operating room. The preparation of the coating process included the prepolymer solution as described above, antibiotic (rifampin), a human intramedullary hip implant, spray bottle or paintbrush, and a UV light source. Rifampin (Rif) and the prepolymer solution were mixed together and then transferred to the spray bottle. The human intramedullary hip implant was either sprayed or painted by brush with the PEG-PAM/Rif polymer solution and then irradiated under UV light for 2 min to yield a homogeneous PEG-PAM/Rif coating on the surface of the implant (Fig. 8). This process was easily performed under sterile conditions.

## Discussion

Infection remains one of the most challenging and devastating complications of orthopedic implant surgery, resulting in a considerable burden to both patients and the healthcare system. Outside of explantation, few options currently exist to combat the formation of biofilm on metallic implant surfaces besides traditional intravenous antibiotic therapy, which remains the mainstay of treatment despite systemic toxicity limiting dosing. Although primitive solutions to deliver high concentrations of antibiotics locally exist, such as antibiotic powder application and antibiotic-impregnated PMMA cement, they possess inherent and significant flaws which make them far from an ideal solution.

The concept of antimicrobial implant coatings has consequently been explored as a method to protect the surface of the implant from bacterial colonization and locally deliver antimicrobials at high concentrations for extended durations; however, no implant coating has made it to market to date. Existing technologies such as iodine and nanosilver implant coatings have been unable to demonstrate (i) in vivo sustained controlled release above minimum inhibitory concentrations, (ii) safety with no detrimental effect on osseointegration, and (iii) the avoidance of selection pressures leading to bacterial resistance. These technologies have also led to procedural concerns, as coating an implant during manufacturing reclassifies it as a drug-delivery device, affecting the shelf life of the implant thus imposing different federal regulatory requirements. To date, with no coatings available and no proven effective powder or bead mechanism, there still exists no standard of care delivery system for local antibiotic therapy in America. Given this clinical problem, novel strategies are needed to decrease the risk of implant-related infection utilizing safer and more reliable methods of local antibiotic delivery.

Herein, we developed a unique polymer formulation to carry antimicrobial compounds with a single layer, rapid polymerization design. The copolymer PEG-PAM requires just a few minutes to coat the implant and has demonstrated antimicrobial efficacy surpassing PEG-PPS. Our study demonstrates that a variety of antibiotics such as vancomycin, ceftriaxone, cefazolin, cefepime, tobramycin, piperacillin, and tazobactam, clindamycin, linezolid, rifampin, and various combinations of these antibiotics can be encapsulated in the PEG-PAM coating without losing their efficacy. In addition, these antibiotics can be passively released over 2 weeks to maintain the MIC during the perioperative period. The local delivery of antibiotics resulted in a significant decrease in bacterial burden as measured by in vivo bioluminescence imaging. Also, the isolated CFUs from implants and surrounding tissue on POD 21 confirmed successful delivery of antibiotics, as vancomycin-loaded PEG-PAM prevented 100% bacterial colonization on the implant in the model of PJI. It is important to note that while the eradication of established biofilm infections requires several times the MIC of a given antibiotic, the purpose of our coating is to prevent bacterial colonization of the implant in the early postoperative period. During this time bacterial inoculums are generally low, and therefore 2 weeks above the MIC would protect the implant in the vast majority of cases.

With any othopaedic implant coating, it is important to consider the ability of the coating to withstand the process of implantation. In orthopaedic surgery, several different methods are used to achieve bone/implant fixation including compliant/compressive osseointegration using a press-fit technique, cross pins, screws, and grout fixation. Of these, only the process of press-fit insertion (which only applies to a subset of all orthopaedic implants and procedures) is likely to deliver a significant shear force to the coating. While the data presented herein does not prove that the coating is able to withstand any shear force, it does suggest that a majority of the surface abrasions to the coating, if experienced, are unlikely to be clinically significant in most scenarios. It is possible that if a coated implant is forced into a canal that is under-reamed, some of the coatings will be removed. However, the SEM images we obtained following reaming and press-fit implantation of coated pins into a Sawbones bone mimic as well as mouse cadaver femurs showed the coating to be largely intact and able to withstand the majority of the shear forces it experienced. This qualitative understanding of the coating post-implantation is further strengthened by our in vivo data showing the ability of the PEG-PAM/antibiotic coating to protect 100% of the implants from bacterial colonization in the PJI model.

With the different strategies for bone/implant fixation listed above in mind, we set out to determine the effect of the PEG-PAM/Vanc coating on osseointegration. Our in vivo osseointegration experiments showed that there was delayed osseointegration in the PEG-PAM/Vanc coated implants compared to the control group at 2 weeks, but no difference between the groups at 4 weeks post-implantation. This is consistent with our data showing that the coating is largely resorbed by 3 weeks. Importantly, while osseointegration may be delayed in the early post-operative period while the coating is still present, we have shown that it is not inhibited after the PEG-PAM/Vanc coating is resorbed. In clinical orthopaedic surgery, osseointegration is an important feature of long-term implant stability for implants that employ this fixation strategy. However, osseointegration is not a prerequisite for structural stability and weight-bearing. Patients who undergo total hip replacements are encouraged to bear full weight on the operative extremity immediately after surgery. This is true despite the fact that osseointegration has not yet occurred on the first day after surgery, a process that takes up to 6–8 weeks in humans^36^. While further study is required to evaluate the effect of the PEG-PAM/antibiotic coating on the osseointegration of load-bearing implants in humans, it is not necessarily the case that a delay in early postoperative osseointegration would have any effect on outcomes. With that in mind, an alternative strategy that would preclude the concerns of both the shear stress on the coating during implantation and its effect on osseointegration would be to only coat the intercalary segments of the implant and leave the intraosseous portions of the implants uncoated. In such a scenario, the portion of any implant that is not press-fit into bone but which faces towards and is within the joint would be covered with the PEG-PAM/antibiotic coating. Such a strategy would be especially beneficial in the setting of mega-prostheses (i.e. distal and total femur replacements) where only a very small portion, if any, of the implant is fixed within the bony canal. Since the coating is antibiotic eluting, this would create an antibacterial milieu that would offer significant protection against bacterial colonization even if not every portion of the implant is covered.

The reasons for the differing efficacy of the PEG-PAM/Vanc coated pins in our model of PJI compared to our model of spine implant infection are unknown. However, it is possible that the intrinsic differences between the local environments of the surgical beds in the knee joint compared to the spine may contribute. The spine implant lies within a highly vascular wound bed which is non-encapsulated, while the knee joint is within an enclosed, synovial joint with relatively less vascularity. This results in two different microenvironments in which the bacteria are growing and the coating is being tested. In addition, in the spine, a 0.1 mm diameter stainless steel implant is used, while in the joint a 0.8 mm diameter titanium implant is used. It is possible that the PEG-PAM/Vanc coating was affected by the difference in the implant size or composition. Yet with minor modification to PEG-PAMDA/Vanc, the efficacy of the coating was considerably improved in the spine implant infection model, highlighting the potential versatility of this coating in the clinical setting.

The thiol-ene “click” reaction has been widely employed as a powerful method of polymerization in polymer chemistry and materials science such as polymer networks formation, bioconjugation, and surface modification, among others^37^. One significant advantage of thiol-ene “click” chemistry compared to other “click” chemistries is the ability to use light to achieve spatial and temporal control of the reaction, thereby greatly expanding the application to fields such as dental curing materials and 3D-printing resins^38^.

Although numerous coating techniques have been developed with varying degrees of in vitro and in vivo efficacy in preventing biofilm infections^39–41^, to our knowledge, the coating described in this study is the first technique that allows for rapid application within a short time frame acceptable to the surgeon and patient in the operating room immediately before implantation. Indeed, due to the widely accepted “click” feature of our coating process utilizing UV light, we have demonstrated that our coating technique can be performed in mere minutes, rendering it practical and translatable to the operating room. Moreover, through our unique coating process, we demonstrated the ability to mix various antibiotics with the photopolymer solutions, providing necessary antimicrobial compatibility in order to treat various bacterial infections. Based on pre-operative cultures and susceptibility testing, the individual physician could potentially choose to incorporate various antibiotics that are expected to perform best for the individual patient at the point-of-contact in the operating room. While the point-of-care nature of the PEG-PAM/antibiotic coating has several benefits, it does raise the issue of quality control and reproducibility. However, there are several precedents for the optimization of complex intra-operative point-of-care mixtures including antibiotic-loaded bone cement^42^.

Additionally, we would like to highlight the fact that while many polymers have demonstrated efficacy against static endpoints of histology and CFUs, few have demonstrated efficacy in a model of longitudinal non-invasive tracking of infection. This is an essential distinction with respect to the clinical challenge faced in orthopaedic surgery as demonstrating fewer colony-forming units at a given time point is far easier than demonstrating suppression of the downstream effects of biofilm and osteolysis. In fact, our group and others have published extensively demonstrating the inability of static assays (histology, CFUs) to assess the translational viability of a coating^9,43–45^. These less stringent in vivo assays are a fundamental reason that no coating has come to market to date despite a plethora of successful preclinical data.

In addition to regulatory hurdles, there is a legal issue of liability raised by an intraoperative coating. If a surgeon modifies an implant with a coating in the operating room, a legal “gray” area of who assumes responsibility for the implant’s functioning arises (i.e. does the hospital or physician become liable for implant-related failures). This is a common issue in orthopaedic surgery as implants are “modified” frequently to address specific clinical needs. Whether adding antibiotic-impregnated bone cement or bending stainless steel or titanium plates to better contour to bone, or using a metal cutting burr to shorten a stem, implants are often permanently modified to address intraoperative challenges. Implant manufacturers have retained responsibility for their devices despite this, assuming the mode of failure was not directly related to the modification (i.e. an articular wear issue like polyethylene debris is unrelated to a modification of the stem with polymethylmethacralate). Along these lines, this transient polymer coating, completely absorbed by 3 weeks, would provide little legal cover for implant failures having to do with long-term wear, metal fatigue fracture, or poor functioning.

There are several important limitations in this study. First, this coating is designed to deliver antibiotics for only a short time period (2 weeks) and therefore would not protect against late-onset infections from processes such as skin breakdown or hematogenous spread. However, as the coating process presented here is a rapid single-layered deposition, the addition of further layers of polymers producing a longer duration of antibiotic elution is easily achievable without sacrificing the practicability of this technology. Further studies are underway to assess the details of longer duration from additional coating layers. Second, in this study, we evaluated the efficacy of this coating against *S. aureus* in vivo, the most common clinical pathogen in orthopedic implant infections^8^. Additional clinically relevant bacterial strains should be examined, such as *S. epidermidis, Propionibacterium acnes*, and *methicillin-resistant S. Aureus* (MRSA). Third, our in vivo efficacy studies are limited by the limitations of any small animal model, which simplifies the surgical procedure dramatically. However, the benefits of the model allow for rapid feedback from experiments and host manipulation. Moving forward, further evidence from preclinical investigations in larger animals and humans would be needed. Finally, even though we demonstrated that our coating process on a human intramedually hip implant can be rapidly achieved in the operating room, for a commercial implant coating process, it may be necessary to develop a specialized UV coating device. Such a device would need to enable fast and efficient three-dimensional delivery of photons homogenously on the surface of large implants in a sterile manner.

In summary, we developed an efficient in situ polymer coating strategy for metallic implants, enabling the delivery of antibiotics from the polymer coating to prevent bacterial biofilm infections. This rapid polymerization is critical, as it confers the advantages of a rapid point-of-care coating in the operating room, making possible a personalized medicine approach (adding the antibiotics/antimicrobials most appropriate for a specific patient or environment), avoiding issues of limited shelf-life of a drug-delivery device, and achieving cost containment by offering this coating as an adjuvant for high-risk patients rather than an added cost on all implants.

## Methods

### Polymer synthesis and characterization

Synthesis of polyallyl-mercaptan (PAM). 1 mol% DMPA was added to the 1:1 stoichiometric ratio mixture of 1,3 propanedithiol and allylsulfide and then the mixture was placed under 365 nm UV light (20 mw/cm^2^, Omnicure S1000) irradiation for 30 min. The crude product was precipitated in Acetone/Methanol and the white powders were dried under vacuum and then analyzed by 1H-NMR for structural determination. 1H NMR (400 MHz, Chloroform-d) δ 5.79–5.77 (m,1H), 5.08–5.13 (m, 2H), 3.14–3.12 (m, 2H), 2.77–2.48 (m, 40H), 1.94–1.74 (m, 20H).

### PAMDA synthesis

1 mol% DMPA was added to the 1:1.2 stoichiometric ratio mixture of 1,3 propanedithiol and allylsulfide and then the mixture was placed under 365 nm UV light (20 mw/cm^2^, Omnicure S1000) irradiation for 30 min. The crude product was precipitated in Acetone/Methanol and the white powders were dried under vacuum and then analyzed by 1H-NMR for structural determination. 1H NMR (400 MHz, Chloroform-d) δ 5.79–5.77 (m,2H), 5.08–5.13 (m, 4H), 3.14–3.12 (m, 4H), 2.77–2.48 (m, 20H), 1.94–1.74 (m, 10H).

### Oxidation

Polythioether (0.5 g) was dissolved in chloroform (50 mL) at room temperature. Subsequently, 30% hydrogen peroxide solution (0.5 mL) was added to the reaction flask. After 2 h of reaction, the formed polymer was precipitated in cold ether and dried in a vacuum oven at 50 °C.

### FT-IR measurement

FT-IR spectra were collected by Jasco 420 FTIR Spectrophotometer. The sample pellet was prepared by mixing the 10 mg polymer and 500 mg KBr.

### GPC measurement

Gel permeation chromatography/light scattering (GPC/LS) was performed on an SSI Accuflow Series III liquid chromatograph pump equipped with Wyatt DAWN EOS light scattering (LS) and Optilab rEX refractive index (RI) detectors. Separations were achieved using 105, 104, and 103 Å Phenomenex Phenogel 5 µm columns in chloroform. GPC/LS samples were prepared at concentrations of 5 mg/mL.

### Rheology measurement

PEG-PAM (5 wt% in water) hydrogels were allowed to self-assemble for 30 min before transferring to an 8 mm plate-to-plate rheometer (Physica MCR 301, Anton Paar, Ashland, VA). An evaporation blocker system was used during measurements. For frequency sweep, the data were collected for the modulus with a frequency range of 0.1–100 rad/s under a 1% constrain at 25 °C. For amplitude sweep, the data were collected for the modulus with a frequency of 1 rad/s under a constrain of 1% at 25 °C.

### Coating process

Titanium Kirschner (K) wires (0.8 mm in diameter) were submerged in a solution containing a mixture of PAM, star-PEG-thiol (6 wt%), and vancomycin (20 mg/ml). The “wet” titanium wires are irradiated under UV light (365 nm, 20 mw/cm^2^) for 5 min and dried at room temperature to induce the formation of star-PEG-PAM.

### Coating characterization

The surface features and compositional changes of the implant due to the polymer coating were examined using Nova 230 Nano scanning electron microscopy (SEM) and energy dispersive spectroscopy (EDS). Representative images were acquired under standard operation, and EDS analysis was performed under 10.0 kV voltage and at 35.3 degree take-off angle.

### In vitro release kinetics

In vitro release of vancomycin from the coating was conducted by submerging the K-wires in 200 µL of PBS and incubating at 37 °C. The buffer was refreshed daily for at least one week, and the amount of released vancomycin was quantified by a UV-Vis spectrometer based on their UV absorption at 280 nm.

### Zone of inhibition

S. aureus *(*Xen36) was prepared for inoculation in accordance with previously published protocols from this laboratory^9^. Of note, Xen36 can be isolated from contaminants due to possession of a kanamycin resistance selection marker on its lux operon. Xen36 stab cultures were streaked onto Kanmycin-200 μg/ml LB Agar plates (Luria Broth plus 1.5% bacto agar, Teknova) and cultured at 37 °C overnight. Next, single colonies of *S. aureus* were individually grown in Kanmycin-200 μg/ml TSB and again cultured overnight at 37 °C in a shaking incubator (200 rpm) (MaxQ 4450, Thermo). After a 2 h subculture in TSB of a 1:50 dilution of the resultant culture, mid-logarithmic phase bacteria were attained (0.7 OD). Three fresh kanamycin-200 plates (Luria Broth plus 1.5% bacto agar, Teknova) were incubated at 37 °C for 15 min in order to warm agar. Permanent black ink was used to divide the surface area of each plate into 3 equal parts. 100μL of mid-logarithmic phase Xen36 was pipetted onto each plate and spread evenly. Three uncoated pins, three PEG-PAM pins, and three PEG-PAM/Vancomycin-loaded pins were placed in the middle of each quadrant. Kanamycin-200 (Luria Broth plus 1.5% bacto agar, Teknova) plates were incubated overnight at 37 degrees Celsius. Plates were removed and the diameter of each zone of inhibition was measured.

### In vitro assessment of antibacterial properties of coatings

The coating process was the same as the previously mentioned coating process but with different antibiotics other than vancomycin. The other 8 pharmacy available antibiotics (Ceftriaxone, Cefazolin, Cefepime, Tobramycin, Piperacillin and Tazobactam, Clindamycin, Linezolid, and Rifampin) were loaded into the PEG-PAM coating at the concentration of 20 mg/ml in the dipping solution. After the UV coating process, samples were placed in separate wells in a 96-well plate and 100 μl of TSB was added to each well. Fresh overnight liquid culture of bioluminescent *S. aureus* Xen 36 was diluted to 1 × 10^4^ CFU ml^−1^ and 100 μl of the liquid culture was added into separate wells. All samples were incubated at 37 °C. Bioluminescence was measured at 0 h, 2 h, 8 h, 24 h, 48 h after exposure to either the control group or the antibiotics-loaded group by IVIS Lumina II in vivo imaging system (PerkinElmer, Hopkinton, MA).

### In vivo osseointegration evaluation

All animal procedures were approved by the UCLA Animal Research Committee (ARC #2008-112-41 C). To evaluate the effect of the PEG-PAM/Vanc coating on osseointegration, push-in-force measurements were used. 12-week-old male C57BL/6 wild-type mice were used in all experiments (Jackson Laboratories). The distal femur of 24 mice were implanted with an untreated control Ti implant and 24 mice were implanted with a Ti implant coated with PEG-PAM/Vanc. At 2- and 4-weeks following implantation, 12 mice from each group were euthanized and the femurs were harvested. Each femur was then embedded vertically in a mechanical testing block using methylmethacrylate resin so that the distal, flat end of the implant was exposed. The mechanical withholding strength was measured by pushing the implant in a retrograde fashion into the femoral canal using a custom-made stainless-steel pushing rod mounted on a 1000 N load cell (Instron, Canton, MA). The axial load on the implant was applied at a crosshead speed of 1 mm/min, and the maximum load (N) to displacement was measured as the implant push-in value.

### Mechanical stability of PEG-PAM/antibiotic coating

Two distinct methods were used to qualitatively evaluate the mechanical stability of the PEG-PAM/Vanc coating following press-fit implantation. In each case, 6 Ti implants were coated with PEG-PAM/Vanc. The first method employed a synthetic bone mimic (10 PCF polyurethane foam block, Sawbones). We compressed the flat surfaces of two Sawbones blocks together using lag screws. The potential space between the two blocks was then reamed first with a 25- and then a 21-gauge needle as per our implantation protocol and the coated implants were inserted by hand (Fig. 4f, Supplementary Fig. 7). Following insertion, the screws in the blocks were removed, the blocks separated, and the implants carefully lifted from the blocks and imaged via scanning electron microscopy (SEM). Using this method, the force on the coating was only felt during insertion.

The second set of 6 coated implants were press-fit in a retrograde fashion into freshly harvested cadaver mouse femurs, and then pulled out from the direction in which they had been inserted (Supplementary Fig. 8). Following insertion and removal, the implants were imaged via SEM. The insertion site in the intercondylar notch was sequentially reamed first with a 25- and then a 21-gauge needle prior to implant insertion as per the implantation protocol (Supplementary Fig. 9). Unlike the Sawbones technique described above, in the cadaver femur technique the coating experienced both the force of insertion and removal.

### In vivo assessment of PEG-PAM coating in PJI model

All animal procedures were approved by the UCLA Animal Research Committee (ARC #2008-112-41 C). The bioluminescent *Staphylococcus aureus* Xen36 strain contains a bioluminescent lux operon construct integrated into a stable bacterial plasmid that naturally produces a blue-green light emitted only by metabolically active bacteria. It has previously been demonstrated to be optimal for use in this established mouse model of arthroplasty infection and was grown and cultured as previously described^9^. 12-week-old male C57BL/6 wild-type mice were used in all experiments (Jackson Laboratories). To model an orthopedic implant infection, one of either a medical-grade 0.8 mm diameter K-wire titanium implant, or a 0.1 mm, 0.6 mm, or 0.8 mm diameter K-wire stainless steel implant, either not coated, pre-coated with PEG-PAM, PEG-PAM with Vancomycin, PEG-PAM with Rifampin, or PEG-PAM with Vancomycin and Rifampin was surgically placed into the right distal femur of mice and the joint was challenged with Xen36. Mice were anesthetized via inhalation of isoflurane (2%) and in vivo bioluminescence imaging was performed by using the IVIS Lumina II in vivo imaging system (PerkinElmer, Hopkinton, MA)^9^. The bioluminescence signals were measured on POD 0, 1, 3, 5, 7, 10, 14, and 21. To confirm that the bioluminescence signals corresponded to the bacterial burden in vivo, bacteria adherent to the implants and surrounding tissue were quantified through sonication and CFU counting. High-resolution radiographs were performed on POD 0, 7, 14, 21, and 28 to qualitatively assess osseo-integration and bone resorption. All radiographs were performed using a Faxitron^®^ LX-60 Cabinet radiography system with a variable kV point projection X-ray source and digital imaging system (Qados, Cross Technologies plc, Berkshire, United Kingdom).

### In vivo assessment of coating in spine surgery model

The following is a summary from the previously published description of the in vivo mouse model of spine implant infection^35^. All procedures were approved by the UCLA Animal Research Committee (ARC #2008-112-41 C). A bioluminescent strain of *S. aureus* (Xen36, PerkinElmer, Hopkinton, MA) was incubated, purified, washed, and diluted to the desired inoculum (1 × 10^3^ colony-forming units [CFU] in 2 ml PBS). Twelve-week-old male C57BL/6 J mice (Jackson Laboratories, Bar Harbor, ME) were subjected to survival surgery in which a midline skin incision was centered over the lower lumbar spine. The fascia was entered over the spinous process of the lower lumbar spine, and the para-spinal muscles were sharply dissected away from their osseous attachments to gain access to the posterior elements of the L4 vertebra. A 0.1 mm diameter L-shaped stainless-steel implant was then transfixed into the L4 spinous process of the lumbar spine. Following placement of the implant, the 1 × 10^3^ CFUs of bioluminescent Xen 36 *S. aureus* is inoculated directly onto the implant. The L-shaped stainless-steel implants were coated with either PEG-PAM or PEG-PAMDA with Vancomycin for two experiments. The first experiment compared PEG-PAM against PEG-PAM with Vancomycin, and the second experiment compared PEG-PAMDA (3 layers) against PEG-PAMDA with Vancomycin (3 layers). The bioluminescence signals were measured on POD 0, 1, 3, 5, 7, 10, 14, 21, and 24.

### Statistical analysis

Statistical analyses were performed in Prism (Graph Pad) using a 95% confidence interval. Analyses were performed using ANOVA, repeated measure ANOVA or Student’s *t*-test as indicated in the figure legend. The number of replicates is also indicated in the figure legends.

### Reporting summary

Further information on research design is available in the Nature Research Reporting Summary linked to this article.

## Supplementary information

Supplementary Information

Reporting Summary

## Data Availability

Raw data for graphs plotted in prism can be found at 10.6084/m9.figshare.14614560. All other raw data and materials will be made available by the corresponding author upon written request. PEG-PAM will be provided through an MTA agreement. Any materials no longer available in the corresponding authors laboratories will not be provided and every effort to provide an alternative source given.
