# Supplementary material for: Point-of-care antimicrobial coating protects orthopaedic implants from bacterial challenge
Source: Nat Commun. 2021 Sep 16;12:5473. doi: 10.1038/s41467-021-25383-z (PMC8445967; doi:10.1038/s41467-021-25383-z)
Supplement: Supplementary file 1 — Supplementary Information [file 41467_2021_25383_MOESM1_ESM.docx]

SUPPLEMENTARY INFORMATION FOR

Point-of-care Antimicrobial Coating Protects Orthopaedic Implants From Bacterial Challenge

**Authors:** Weixian Xi,^1,2†^ Vishal Hegde,^2†^ Stephen D. Zoller,^2†^ Howard Y. Park,^2†^ Christopher M. Hart,^2†^ Takeru Kondo,^3^ Christopher D. Hamad,^2^ Yan Hu,^2^ Amanda H. Loftin,^2^ Daniel O. Johansen,^2^ Zachary Burke,^2^ Samuel Clarkson, ^2^ Kellyn Hori,^2^ Zeinab Mamouei,^2^ Hiroko Okawa,^3^ Ichiro Nishimura,^3^ Nicholas M. Bernthal,^2^*** Tatiana Segura^1,4^***

**Affiliations:**

^1^Department of Chemical and Biomolecular Engineering, University of California Los Angeles

420 Westwood Plaza, 5531 Boelter Hall, Los Angeles, CA 90095, United States

^2^Department of Orthopaedic Surgery, Orthopaedic Hospital Research Center, David Geffen School of Medicine at University of California Los Angeles, Los Angeles, CA 90095, United States

^3^Weintraub Center for Reconstructive Biotechnology, Division of Advanced Prosthodontics, University of California Los Angeles School of Dentistry, Los Angeles, CA 90095, United States

^4^Department of Biomedical Engineering, Neurology, Dermatology, Duke University, 101 Science Drive, Durham, NC, United States

*E-mail of corresponding author: Tatiana.segura@duke.edu，NBernthal@mednet.ucla.edu,

†These authors contributed equally to this work.

**Supplementary Figure legends:**


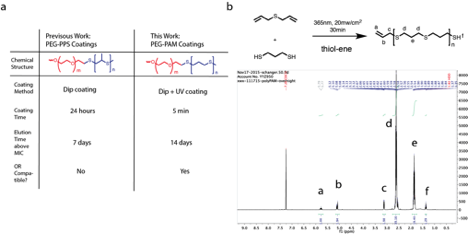


**Supplementary Figure 1**. (A) Generic comparison of previous work PEG-PPS and current work PEG-PAM coating on implant. (B) Synthesis of polyallyl-mercaptan (PAM) from copolymerization of 1,3 propene dithiol and allyl sulfide and ^1^H-NMR identification of PAM in CDCl_3_. 1H NMR (400 MHz, Chloroform-d) δ 5.79-5.77 (m,1H), 5.08-5.13(m, 2H), 3.14-3.12 (m, 2H), 2.77-2.48 (m, 40H), 1.94-1.74 (m, 20H), consistent with a polymerization degree of 10.

**Supplementary Figure 2.** GPC trace of PAM polymer in CHCl_3_. LS is the trace monitored by light scattering detector, RI is the trace monitored by refractive index (RI) detector. Using PEG standards both LS and RI traces reveal a Mn of ~767 MW.

**Supplementary Figure 3**. SPR signal measurement on gold surface with addition of 0.1mg/ml PEG-PAM in methanol. Intensity of reflected light against time for PEG-PAM addition, followed by subsequent water wash on gold surface. The SPR signal plot shows that PEG-PAM can chemisorb to gold.


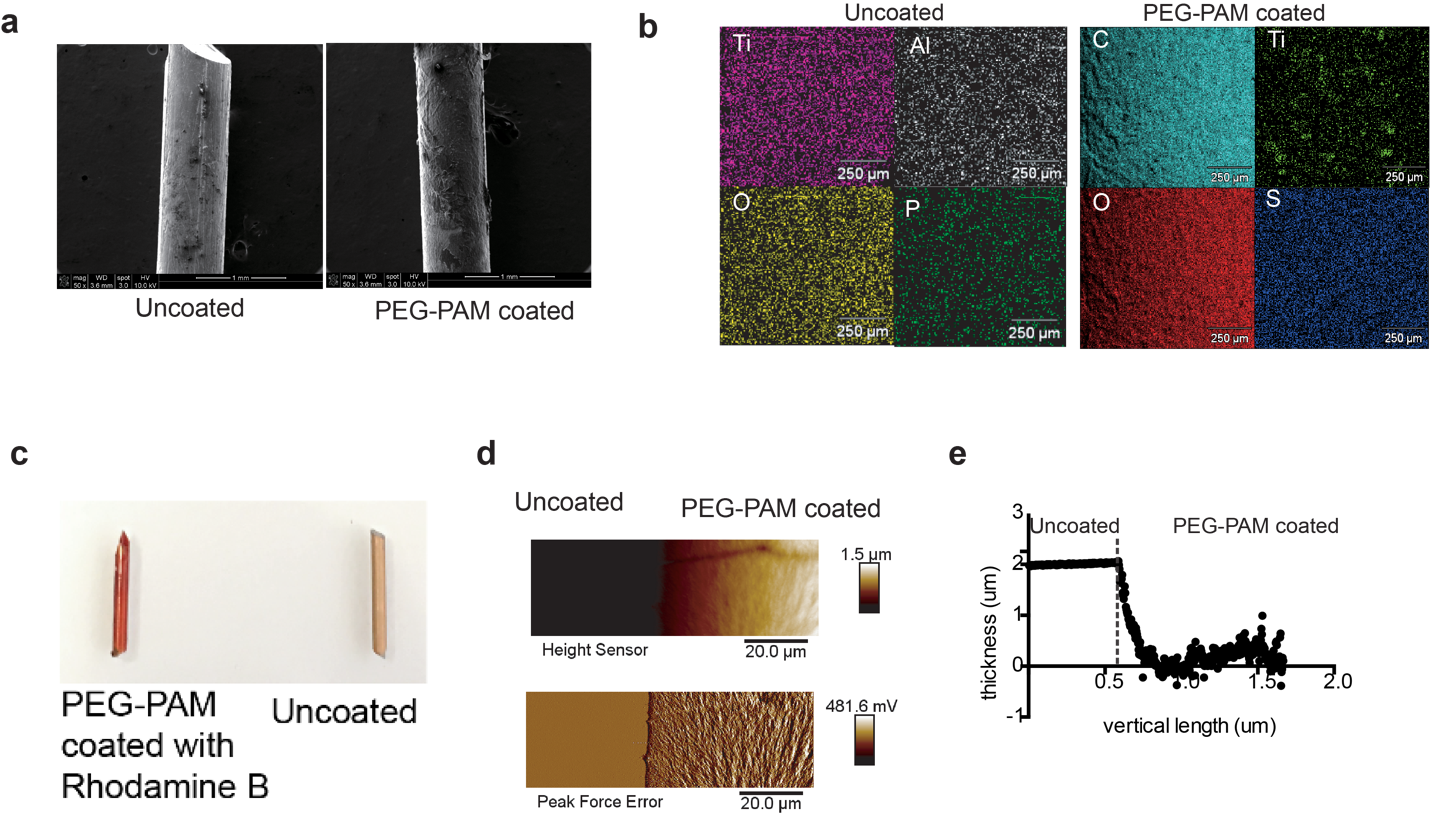


**Supplementary Figure 4.** PEG-PAM coating characterization. (a) SEM image of uncoated and PEG-PAM coated titanium pins show a change in surface roughness as a result of the coating process. (b) Elemental analysis of uncoated and PEG-PAM coated titanium surface show increase in C, O, and S for the coated surfaces. (c) Incorporation of Rhodamine during coating (PEG, PAM, DMPA, Rhodamine-B) result in pins that are visually colored. (e,f) AFM and 3D profilometer scan of PEG-PAM coated titanium surface demonstrates that the coating is uniform.


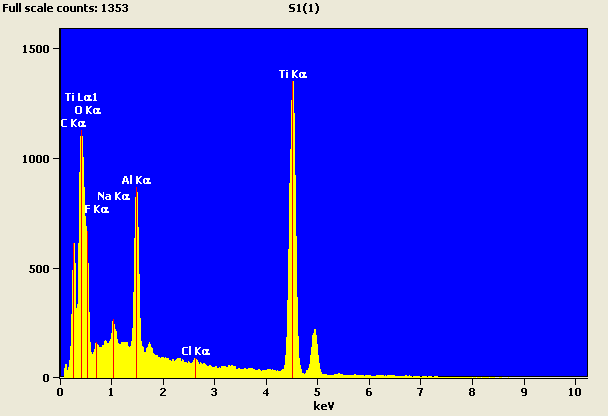


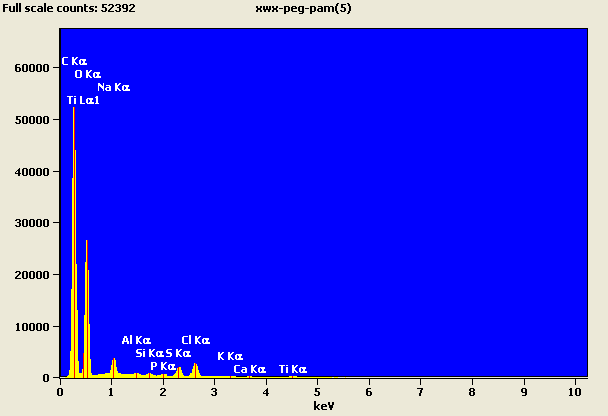


**Supplementary Figure 5**. Energy-dispersive X-ray spectroscopy (EDS) of PEG-PAM coated pins (A) and uncoated pins(B), which represents the distribution of the constituent of the elements on the surface of PEG-PAM coated pins and uncoated pins.

**Supplementary Figure 6**. Vancomycin loading in 2 wt% PEG-PAM coating and 20wt% PEG-PAM coating. The loading amount of vancomycin was calculated from the sum of daily released of vancomycin from 2 wt% PEG-PAM coated pin and 20wt% PEG-PAM coated pin. Measurements were taken from 3 independent samples(n=3). Data is plotted showing the mean and Standard Deviation.


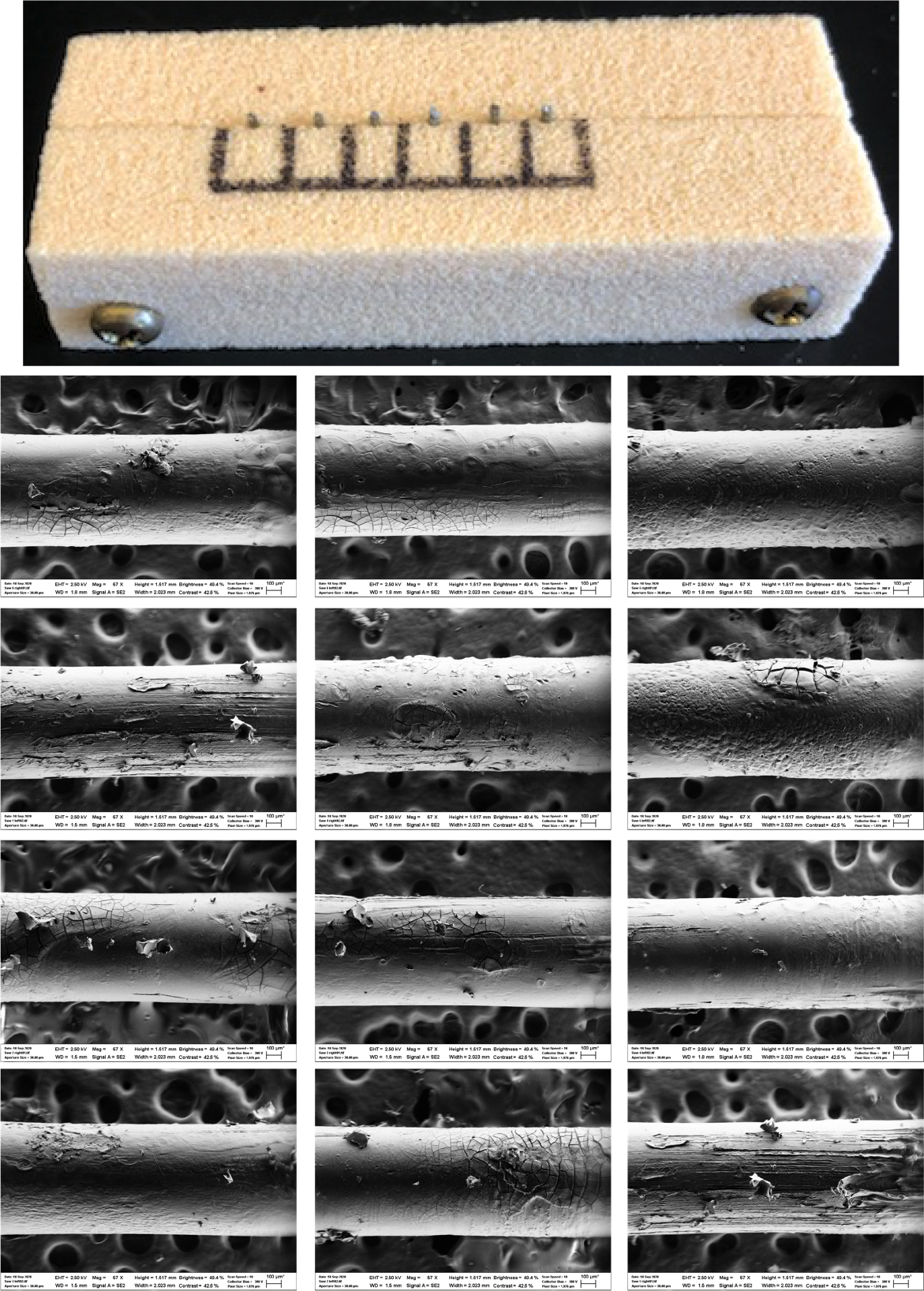


**Supplementary Figure 7.** Mechanical integrity of coating after press-fit insertion into Sawbones bone mimic. Top image shows the block used. 6 pins total, 2 images per pin. All 12 biological replicates are shown.


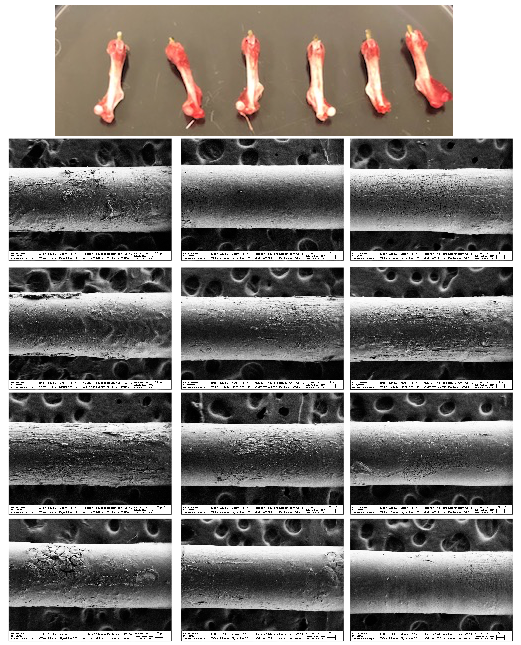


**Supplementary Figure 8**. Mechanical integrity of coating after press-fit insertion and removal from mouse femurs. Top image shows 6 of the femurs used. 6 pins total, 2 images per pin. All 12 biological replicates are shown.


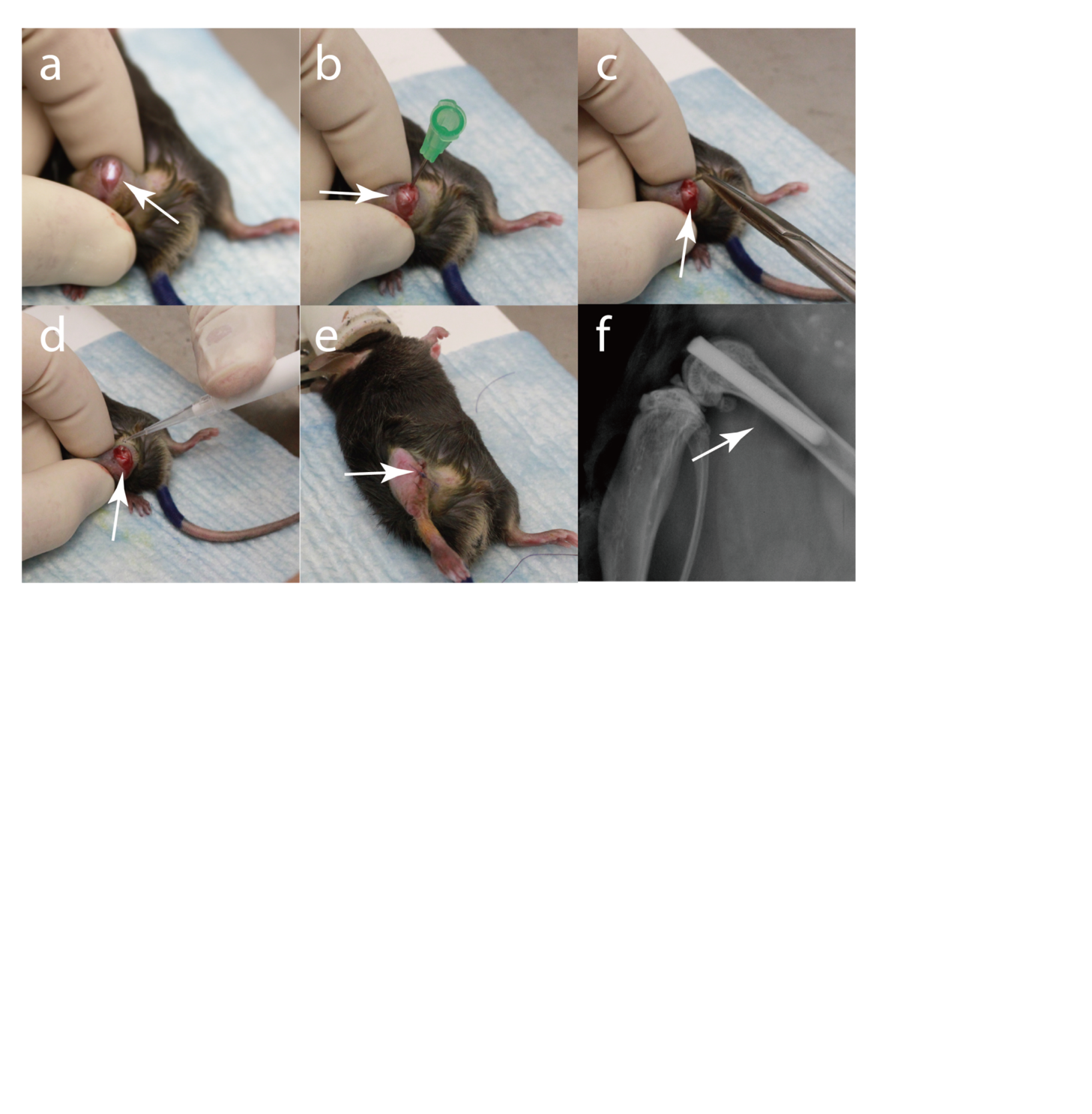


**Supplementary Figure 9**. Surgery procedures for joint replacement model. A skin incision was made over the right knee (a). The distal right femur was accessed through a medial parapatellar arthrotomy with lateral displacement of the quadriceps-patellar complex. After locating the femoral intercondylar notch, the femoral intramedullary canal was manually reamed with a 25-gauge followed by a 21-gauge needle (b). An orthopaedic-grade Kirschner (K)-wire (diameter 0.6 mm) (Synthes) was surgically placed in a retrograde fashion and cut with 1 mm protruding into the joint space (c). An inoculum of S. aureus in 2 ml of normal saline was pipetted into the joint space containing the cut end of the implant (d). The surgical site was closed with Dexon 5-0 sutures (e). A representative radiograph demonstrates the position of the implant with good intramedually fixation of the stem and prominence of the cut surface in the joint (f).


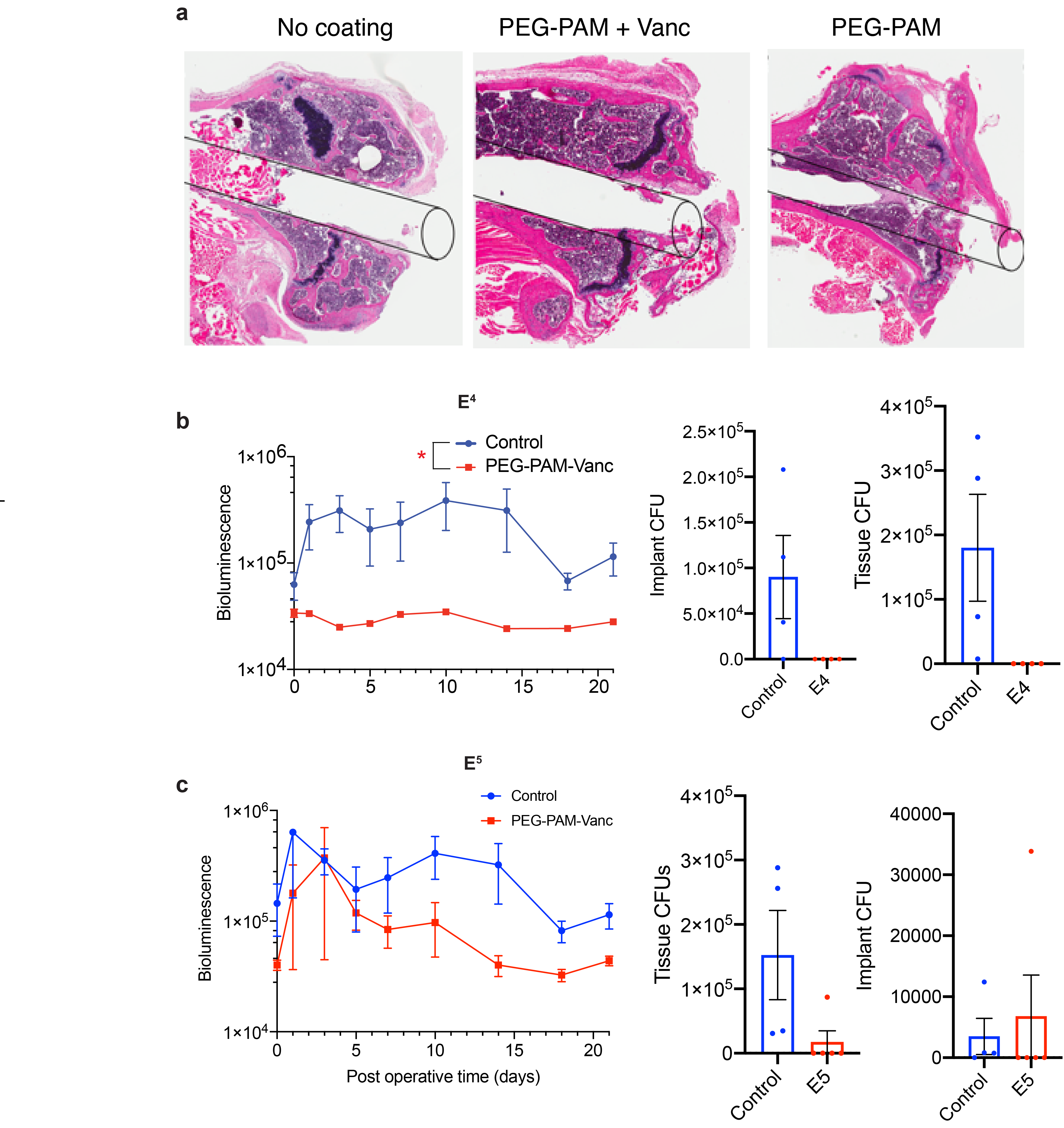


**Supplementary Figure 10.** (a) Histologic sections of No coatings, PEG-PAM, PEG-PAM with vancomycin groups after POD 21. (b) Postoperative *in vivo* S. *aureus* bioluminescence signals with 1.0x10^4^ and 1.0x10^5^,1CFUs loading of bacterial challenge with PEG-PAM+Vanc coating. (n=4 for control group, n=5 for PEG-PAM/Vanc group in 1.0x10^4^ loading group; n=4 for control group, n=6 for PEG-PAM/Vanc group in 1.0x10^5^ loading group. Quantification of colony-forming units (CFUs) cultured from surrounding tissue and implant (n=4 for control group, n=5 for PEG-PAM/Vanc group in 1.0x10^5^ loading group in CFU counting experiment). * are the results from a repeated measure ANOVA indicating statistical significance (p < 0.05) between the indicated groups. Data is plotted showing the mean and Standard Error of the Mean (SEM).
